# Tumor-associated calcium signal transducer 2 expression as a promising therapeutic target in esophageal squamous cell carcinoma: a combined the Cancer Genome Atlas and an institutional cohort

**DOI:** 10.1007/s10388-026-01257-5

**Published:** 2026-09-20

**Authors:** Chiaki Inagaki, Hisato Kawakami, Seiichiro Mitani, Hiroshi Iijima, Ryo Matoba, Akihiko Ito, Osamu Shiraisi, Takushi Yasuda, Hidetoshi Hayashi

**Affiliations:** 1 Department of Medical Oncology, Faculty of Medicine, Kindai University, 1-14-1 Miharadai, Minami-Ku, Sakai-Shi, Osaka, Japan; 2 Genome Medical Center, Kindai University Hospital, 1-14-1 Miharadai, Minami-Ku, Sakai-Shi, Osaka, Japan; 3 Department of Clinical Oncology, Tohoku University Graduate School of Medicine, Sendai, Japan; 4 DNA Chip Research Inc., 3-1200, Shinmaruko-Higashi, KDX Musashikosugi Blgd. 9 F, Nakahara-Ku, Kawasaki-Shi, Kanagaaw, 211-0004 Japan; 5 Department of Medicine/Graduate School of Medical Sciences, Faculty of Medicine, Kindai University, 1-14-1 Miharadai, Minami-Ku, Sakai-Shi, Osaka, Japan; 6 Department of Surgery, Faculty of Medicine, Kindai University, 1-14-1 Miharadai, Minami-Ku, Sakai-Shi, Osaka, Japan

**Keywords:** Esophageal squamous cell carcinoma, TROP2, *TACSTD2*

## Abstract

**Background:**

Esophageal squamous cell carcinoma (ESCC) has a poor prognosis, and new therapeutic targets are required. Antibody–drug conjugates (ADCs) are a promising therapeutic modality that selectively delivers cytotoxic payloads to tumors via cell-surface antigens. Tumor-associated calcium signal transducer 2 (TROP2, *TACSTD2*) has shown therapeutic potential as an ADC target in several malignancies; however, its clinical significance in ESCC remains unclear.

**Methods:**

An institutional cohort of ESCC patients treated at Kindai University was retrospectively evaluated by immunohistochemistry (n=70) and RNA sequencing (n=27). *TACSTD2* copy number alterations, RNA expression, and survival were analyzed using the Cancer Genome Atlas (TCGA) ESCC dataset. IHC was assessed in both resected and biopsy specimens, including metastatic lesions. Survival analyses were performed, stratifying patients by cohort-specific median *TACSTD2* expression levels.

**Results:**

Immunohistochemistry analysis demonstrated uniformly strong TROP2 expression in all evaluable primary and metastatic lesions. In the TCGA dataset, *TACSTD2* mRNA expression was consistently high, irrespective of copy-number status, and was comparable to or higher than that observed in non-small cell lung cancer and breast cancer, tumor types in which TROP2-targeted ADCs are clinically active. The *TACSTD2* high-expression group showed a trend toward worse overall survival in both the institutional and the TCGA cohort, whereas disease-free survival was similar.

**Conclusions:**

TROP2/*TACSTD2* is strongly and consistently expressed in ESCC at the protein and RNA levels, despite infrequent gene amplification, supporting its feasibility as a therapeutic target. High expression shows a trend toward poorer overall survival, warranting validation in larger cohorts.

**Supplementary Information:**

The online version contains supplementary material available at https://doi.org/10.1007/s10388-026-01257-5.

## Introduction

Esophageal cancer is the sixth leading cause of cancer-related mortality worldwide [1]. Esophageal adenocarcinoma is the major histological type in Western countries, whereas esophageal squamous cell carcinoma (ESCC) predominates in Asia, Africa, and South America and accounts for nearly 90% of esophageal cancers in Japan [1, 2]. Approximately half of ESCC cases are unresectable at diagnosis, and most patients treated with curative intent eventually experience recurrence [2].

Systemic chemotherapy is the mainstay treatment for recurrent or metastatic ESCC [3, 4]. Recent phase III trials established anti–PD-1 antibody (nivolumab, pembrolizumab, or tislelizumab) in combination with 5-fluorouracil (5-FU) and cisplatin (CDDP) as a standard first-line treatment for advanced ESCC [5–7]. Dual checkpoint blockade with nivolumab plus ipilimumab is another standard option [6]. Paclitaxel is commonly used for patients with platinum-resistant and/or refractory disease [8]; however, no standard chemotherapy exists beyond platinum- and taxane-based agents, raising an urgent need to uncover future therapeutic targets.

Antibody–drug conjugates (ADCs) are monoclonal antibodies conjugated to cytotoxic agents via chemical linkers that target cell-surface proteins, regardless of the protein’s intrinsic oncogenicity [9]. As a new class of targeted agents, ADCs represent a key area of interest in anticancer drug discovery and development. Tumor-associated calcium signal transducer 2 (TROP2, which is encoded by the tumor-associated calcium signal transducer 2 [*TACSTD2*] gene) is a clinically validated ADC target, with approved agents in select tumor types [10, 11]; however, the clinical relevance of TROP2/*TACSTD2* expression remains poorly characterized in ESCC [12–15]. Therefore, in this study, we first characterized TROP2 protein expression using immunohistochemistry (IHC) in our institutional ESCC cohort. We then evaluated the association between *TACSTD2* mRNA expression and clinicopathological characteristics, as well as patient outcomes, in both the Cancer Genome Atlas (TCGA) ESCC cohort and our institutional cohort.

## Methods

### The Cancer Genome Atlas (TCGA) cohort data collection

The genomic data of primary tumors from the TCGA Pan-Cancer cohort were obtained from the University of California, Santa Cruz Xena data hub (https://xenabrowser.net/datapages/) [16]. Copy number and normalized RNA sequencing (RNA-seq) data (transcripts per million [TPM]) were downloaded. The copy number variation (CNV) was summarized at the gene level using the Genomic Identification of Significant Targets in Cancer 2 (GISTIC2) threshold method and reported as discrete calls (− 2, − 1, 0, 1, 2), representing homozygous deletion, single-copy deletion, diploid, low-level amplification, and high-level amplification, respectively. Clinically relevant data for ESCC samples, including age, sex, history of smoking, drinking habits, pathological stage, and survival, were obtained from the Memorial Sloan Kettering Cancer Center cBioPortal (http://www.cbioportal.org, accessed on September 4, 2025) [17]. Details of the tumor subtypes included in the analysis are summarized in Online Resource 1.

## Patient selection for the institutional cohort

For the institutional cohort, we retrospectively collected clinical data from patients with ESCC and performed genomic and immunohistochemical analyses. The inclusion criteria were as follows: (1) patients with pathologically confirmed ESCC (adenosquamous carcinoma was eligible only when the squamous component accounted for >50% of the tumor area on histopathological review), (2) patients with locally advanced or metastatic ESCC who received systemic chemotherapy and/or radiation at our department between January 2017 and May 2022, and (3) patients with sufficient amounts of archival formaldehyde-fixed paraffin-embedded (FFPE) tumor tissue specimens available for analysis. Consequently, 70 patients were included in this study (Online Resource 2). Clinical data were retrospectively obtained from the electronic records. A person with habitual alcohol drinking was defined as a person who drank ≥60 g of alcohol per day at least 3 days a week. Pathological responses after neoadjuvant chemotherapy and/or radiotherapy were evaluated and graded according to the pathological criteria used in the Japanese Classification of Esophageal Cancer (11th Edition) as follows [18]. Patient characteristics are shown in Online Resource 3.

## Immunohistochemistry (IHC) analysis

Of the 70 patients, 40 had resected specimens and 30 had biopsy specimens for TROP2 IHC evaluation (Online Resource 2). TROP2 IHC analysis of the FFPE specimens was performed, as previously described [19]. Briefly, the FFPE specimens were sectioned at 4 μm, placed on positively charged glass slides, and heated at 60 °C for at least 40 min. Following deparaffinization, antigen retrieval was performed using a Diva Decloaker (BioCare DV2004MX) and blocking was performed. For TROP2 IHC staining, anti-TROP2 rabbit monoclonal antibody (clone A66M7, lot number EB160622-02, 0.5 µg/mL, Eisai) and SignalStain® boost detection reagent (HRP, Rabbit, Cell signaling) were used as a primary and secondary antibodies, respectively. All IHC staining and analyses were performed using Epochal Precision Anti-Cancer Therapeutics. TROP2 IHC results were obtained for all collected FFPE specimens.

## IHC scoring

Each slide was evaluated using HALO® to quantify staining intensity and the proportion of positive tumor cells. Overall TROP2 staining intensity in tumor cells, integrating membranous and cytoplasmic staining, was scored as 0 (no staining), 1+ (weak), 2+ (moderate), or 3+ (strong). TROP2 positivity was defined as ≥75% of neoplastic cells showing 2+ or 3+ intensity.

## RNA sequencing

RNA-seq analysis was performed using surgical FFPE specimens according to the manufacturer’s protocol [20, 21], and results were obtained for 27 of 40 specimens (67.5%). Briefly, total RNA was extracted from two FFPE tissue slices using an miRNeasy FFPE kit (Qiagen) [20]. Whole-transcriptome sequencing was performed using the SMART-Seq Stranded kit (Takara Bio Europe) for library preparation and NextSeq 500, single end 75 bp (Illumina) for sequencing [21]. Sequencing adaptors, low-quality reads, and bases were trimmed using the Trimmomatic-0.39 tool. The sequence reads were aligned to the human reference genome (hg38) using STAR 2.7.9a. The aligned reads were subjected to downstream analyses using the StrandNGS 4.0 software (Agilent Technologies, Santa Clara, CA, USA). The read counts allocated to each gene and transcript (Ensembl Database 2016.12.01) were quantified using the TPM method.

## Outcomes and statistical analyses

The TCGA and the institutional cohort were stratified according to the cohort-specific median *TACSTD2* expression level (high, ≥median; low, <median), and baseline patient characteristics were descriptively summarized. Given the exploratory nature of this biomarker study, we did not perform formal hypothesis testing and therefore did not report P values; instead, we presented effect sizes with confidence intervals (CIs), where appropriate. The 99% CI for the between-group differences in proportions was calculated using the Newcombe–Wilson method to provide a more conservative range across multiple baseline variables. A 99% CI was used for between-group differences in baseline characteristics because a relatively large number of variables were descriptively compared between the TACSTD2 high- and low-expression groups. Survival was estimated using the Kaplan–Meier method, and effect sizes were summarized as hazard ratios (HRs) with 95% CIs using Cox proportional hazards models. Overall survival (OS) was defined as the time from the initiation of primary cancer treatment to death, whereas disease-free survival (DFS) was defined as the time from the completion of primary curative treatment to disease progression or death. In the TCGA cohort, analyses were restricted to patients with available DFS data (n=59), and in the institutional cohort, analyses were restricted to patients with available RNA expression data. Statistical analyses were performed using GraphPad and RStudio [22].

## Results

### Tumor-associated calcium signal transducer 2 IHC staining in the institutional cohort

**Fig. 1:**
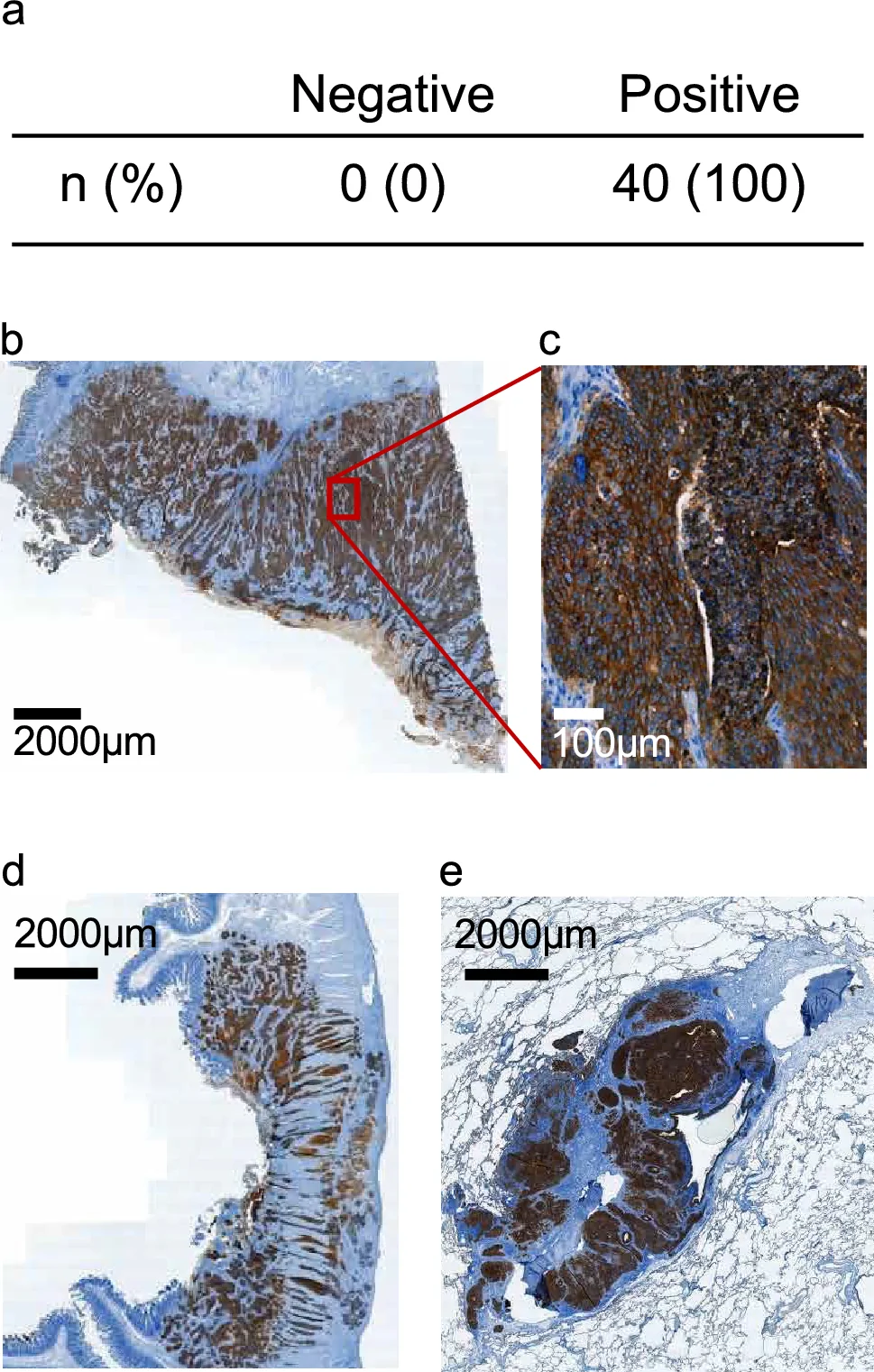
Immunohistochemical analysis of TROP2 expression in ESCC. **a** Percentages of TROP2-positive tumor area across 40 resected ESCC cases, classified as strong (3 +), moderate (2 +), or weak (1 +) positive staining. All cases were TROP2 positive (100%). **b**–**c** Representative low- and high-magnification images of surgically resected primary ESCC showing strong TROP2 staining. **d**–**e** Images of surgically resected metastatic lesion from small intestine **d** and lymph node **e** evaluated TROP2 IHC staining at low magnification. Tumor cells exhibit strong TROP2 staining in the both lesion. ESCC; esophageal squamous cell carcinoma, TROP2; trophoblast cell-surface antigen 2

As high and homogeneous expression of the target antigen on tumor cells is widely considered crucial for optimal ADC efficacy [9], we first investigated TROP2 expression by IHC using all samples in the institutional cohort. All surgically resected samples were positive, with strong staining (40/40, 100%, Fig. 1a–c). Notably, three samples from metastatic sites were included in our cohort, and all showed strong staining (Fig. 1d, e). In addition, all biopsy samples showed strong staining **(**negative 0/30, 0%; positive 30/30, 100%; Online Resource 4a–c).

## *TACSTD2* copy number and mRNA expression value in the TCGA cohort

**Fig. 2:**
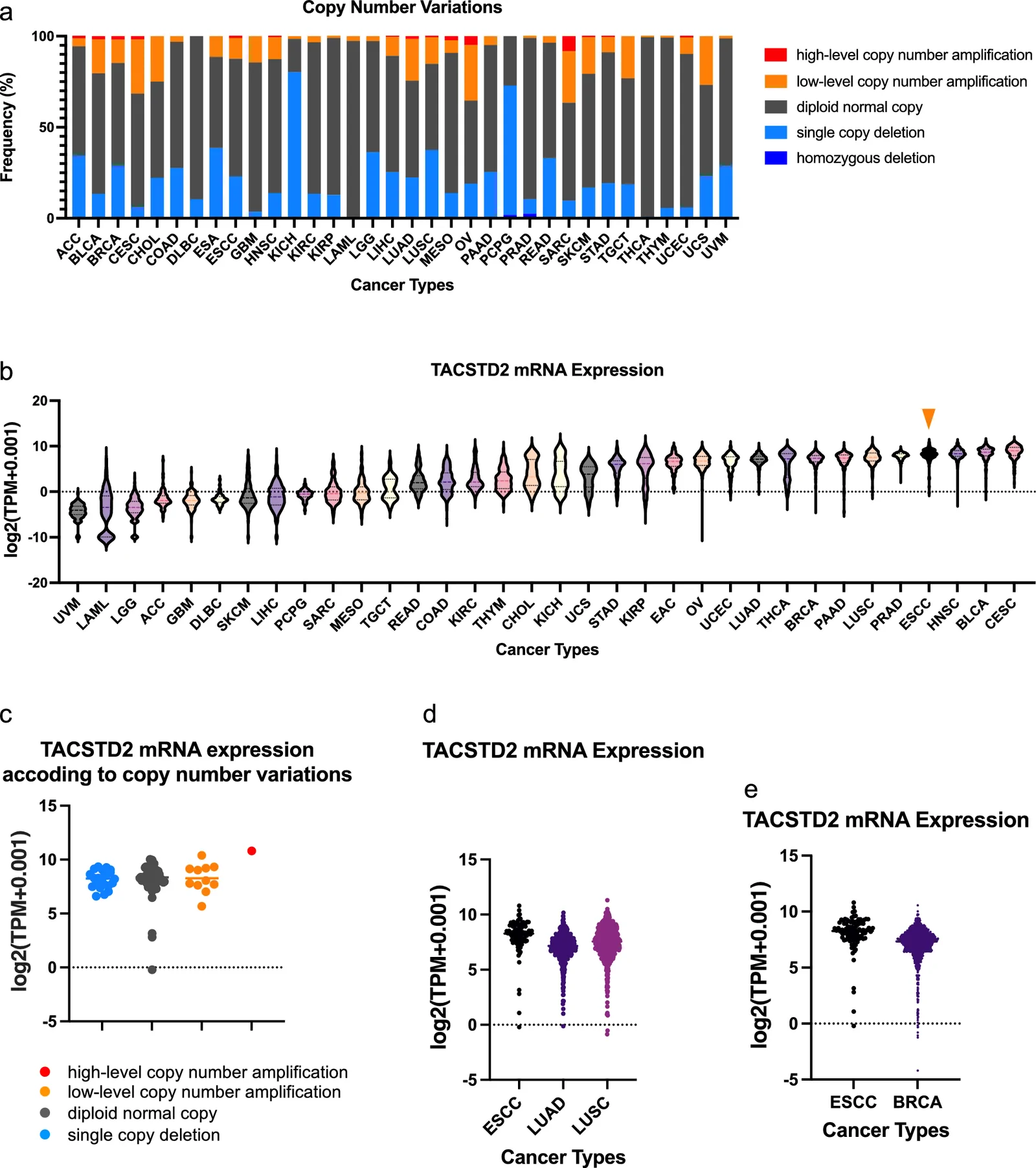
Genomic alterations and expression of *TACSTD2* across cancer types in TCGA. **a** Distribution of *TACSTD2* copy number variations across multiple cancer types in TCGA. Frequencies of high-level amplification (red), low-level amplification (orange), diploid/normal copy (gray), single copy deletion (light blue), and homozygous deletion (blue) are shown. **b** Violin plots depicting *TACSTD2* mRNA expression (log2[TPM+0.001]) across cancer types. **c** Association between *TACSTD2* mRNA expression and copy number variations in ESCC. **d** Comparison of *TACSTD2* mRNA expression between ESCC, LUAD, and LUSC, showing higher expression in ESCC. **e** Comparison of *TACSTD2* mRNA expression between ESCC and BRCA, showing higher expression in ESCC. *BRCA* breast invasive carcinoma, *ESCC* esophageal squamous cell carcinoma, *LUAD* lung adenocarcinoma, *LUSC* lung squamous cell carcinoma, *mRNA* messenger ribonucleic acid, *TACSTD2 * tumor-associated calcium signal transducer 2, *TCGA* the cancer genome atlas, *TPM* transcripts per million

As *TACSTD2* expression in ESCC has not been extensively explored, we next evaluated *TACSTD2* amplification and mRNA expression values using the TCGA dataset. The amplification of *TACSTD2* was infrequently observed across tumor types (Fig. 2a). The median mRNA expression level of *TACSTD2* in ESCC was comparatively elevated among various tumor types (Fig. 2b). *TACSTD2* amplification was not frequently detected in patients with ESCC (high-level copy number: 1/94, 1.1%; low-level copy number: 11/94, 11.7%), whereas *TACSTD2* expression was consistently observed across cases irrespective of copy number status (Fig. 2c). *TACSTD2* expression in ESCC was comparable to or higher than that observed in tumor types, where TROP2-targeted ADC showed clinical efficacy, such as in breast cancer (BRCA) and non-small cell lung cancer (NSCLC, Fig. 2d, e).

## Impact of *TACSTD2* mRNA expression on clinical characteristics and outcomes

TROP2 IHC staining in our institutional cohort was uniformly positive in all samples, thereby preventing stratification based on protein expression. Therefore, we finally evaluated survival outcomes in patients with ESCC in both the TCGA and our institutional cohorts, stratified according to the cohort-specific median *TACSTD2* mRNA expression level (high, ≥median; low, <median). Baseline characteristics are summarized in Tables 1 (TCGA) and 2 (institutional) and were broadly similar between the groups, although some imbalances were observed.

**Table 1:** Characteristics of patients with ESCC from the TCGA

Patients’ characteristics	High (n=46)	Low (n=46)	Difference in proportion High – low [99% CI]
Age, median (range), years	57.5 (36–84)	57 (36–89)	–
Ethnicity, n (%)			
Caucasian	19 (41.3)	22 (47.8)	−6.5 [− 31.9, 19.8]
Asian	21 (45.7)	22 (47.8)	−2.2 [− 28.0, 24.0]
Others	6 (13.0)	2 (4.3)	8.7 [− 8.4, 27.1]
Sex, n (%)			
Male	39 (84.8)	39 (84.8)	0.0 [− 20.7, 20.7]
Female	7(15.2)	7 (15.2)	0.0 [− 20.7, 20.7]
Smoking history, n (%)			
Yes	33 (71.7)	25 (54.3)	17.4 [− 8.7, 4.1]
No	13 (28.3)	18 (39.1)	−10.9 [− 35.1, 14.6]
NA	0 (0)	3 (6.5)	−6.5 [− 22.5, 6.6]
Habitual drinking, n (%)			
Yes	37 (80.4)	30 (65.2)	15.2 [− 9.0, 38.1]
No	8 (17.4)	15 (32.6)	−15.2 [− 37.7, 8.4]
NA	1 (2.2)	1 (2.2)	0.0 [− 14.4, 14.4]
Histology, n (%)			
Squamous	46 (100)	46 (100)	0.0 [− 12.7, 12.7]
Pathological stage, n (%)			
I–II	28 (60.0)	32 (69.5)	−8.7 [− 33.2, 16.8]
III–IV	16 (34.7)	14 (30.4)	4.3 [− 20.7, 28.9]
NA	2 (4.3)	0 (0)	4.3 [− 8.7, 19.5]
cT stage, n (%)			
1–2	22(47.8)	16(34.8)	13 [− 13.4, 37.8]
3–4	22 (47.8)	30 (65.2)	−17.4 [− 41.8, 9.3]
NA	2 (4.3)	0 (0)	4.3 [− 8.7, 19.5]
cN stage, n (%)			
Negative	21 (45.7)	31 (67.4)	−21.7 [− 45.6, 5.0]
Positive	22 (50.9)	15 (32.6)	15.2 [− 11.1, 39.6]
NA	3 (6.5)	0 (0)	6.5 [− 6.6, 22.5]
cM stage, n (%)			
0	41 (89.1)	39 (84.8)	4.3 [− 15.4, 24.3]
1	2 (4.3)	2 (4.3)	0.0 [− 15.8, 15.8]
NA	3 (6.5)	5 (10.9)	−4.3 [− 22.6, 13.3]

*CI* confidence interval, *ESCC* esophageal squamous cell carcinoma, *NA* not available

**Table 2:** Characteristics of the patients in the institutional cohort with surgically resected specimens and transcriptomic data available

Patients' characteristics	High (n=13)	Low (n=14)	Difference in proportion High – Low [99% CI]
Age, median (Min, Max), years	69 (49–81)	67 (56–79)	–
Sex, n (%)			
Male	8(61.5)	12 (85.7)	−24.2 [− 62.1, 20.9]
Female	5(38.5)	2 (14.3)	24.2 [− 20.9, 62.1]
Smoking history, n (%)			
Yes	11 (84.6)	9 (64.3)	20.3 [− 25.1, 58.4]
No	2 (15.4)	5 (35.7)	−20.3 [− 58.4, 25.1]
Habitual drinking, n (%)			
Yes	9 (69.2)	12 (85.7)	−16.5 [− 55.8, 26.8]
No	4 (36.4)	2 (14.3)	16.5 [− 26.8, 56.8]
Sample site, n (%)			
Primary	12 (92.3)	13 (92.9)	−0.5 [− 38.5, 36.0]
Metastatic site	1 (7.7)	1 (7.1)	0.5 [− 36.0, 38.5]
Primary tumor localization, n (%)			
Cervical	2 (15.4)	1 (7.1)	8.2 [− 30.2, 46.7]
Thoracic	10 (76.9)	11 (78.6)	−1.6 [− 43.8, 40.1]
Abdominal	1 (7.7)	2 (14.3)	−6.6 [− 43.9, 33.1]
Histology, n (%)			
Squamous	12 (92.3)	13 (92.9)	−0.5 [− 38.5, 36.0]
Adenosquamous	1 (7.7)	1 (7.1)	0.5 [− 36.0, 38.5]
Clinical stage; cStage, n (%)			
I–II	2(15.4)	2 (14.3)	1.1 [− 38.4, 41.5]
III–IV	11(84.6)	12 (85.7)	−1.1 [− 41.5, 38.4]
cT stage, n (%)			
1–2	2(15.4)	1 (7.1)	8.2 [− 30.2, 46.7]
3–4	11(84.6)	13 (92.9)	−8.2 [− 46.7, 30.2]
cN stage, n (%)			
Negative	0 (0)	3 (21.4)	−21.4 [− 53.2, 17.2]
Positive	13 (100)	11 (78.6)	21.4 [− 17.2, 56.2]
cM stage, n (%)			
0	11 (84.6)	12 (85.7)	−1.1[− 41.5, 38.4]
1; No.104 or 101 alone/other,	2 (15.4)	2 (14.3)	1.1 [− 38.4, 41.5]
No.104 or 101 alone	0 (0)	1 (7.1)	−7.1 [− 41.7, 28.9]
Other	2 (15.4)	1 (7.1)	8.2 [− 30.2, 46.7]
Prior treatment with curative intent, n (%)			
Yes	12 (92.3)	13 (92.9)	−0.5 [− 38.5, 36.0]
Chemotherapy*	12 (92.3)	12 (85.7)	6.6 [− 33.1, 43.9]
Radiation*	1 (7.7)	2 (14.3)	−6.6 [− 43.9, 33.1]
Resection (including ESD)*	11 (84.6)	12 (85.7)	−1.1 [− 41.5, 38.4]
R0–1	9 (69.2)	11 (78.6)	−9.3 [− 50.8, 34.4]
R2	2 (15.4)	1 (7.1)	8.2 [− 30.2, 46.7]
Pathological response of neoadjuvant therapy, n (%)			
0	3 (23.1)	1 (7.1)	15.9 [− 24.3, 54.0]
1	6 (46.2)	10 (71.4)	−25.3 [− 64.0, 23.2]
2	2 (15.4)	1 (7.1)	8.2 [− 30.2, 46.7]
Pathological stage, n (%)			
I- II	4 (30.8)	4 (28.6)	2.2 [− 41.3, 45.6]
III- IV	7 (53.8)	8 (57.1)	−3.3 [− 47.7, 42.2]
Pattern of recurrence			
Local	2(15.4)	2 (14.3)	1.1 [− 38.4, 41.5]
Distal	11(84.6)	12 (85.7)	−1.1 [− 41.5, 38.4]
Number of recurrence sites			
1–2	9 (69.2)	14 (100)	−30.7 [− 65.4, 7.7]
3≦	4 (30.8)	0 (0)	0.30 [− 7.2, 65.4]
Treatment for recurrent/metastatic disease, n (%)			
Yes	13 (100)	14 (100)	0 [− 34.6, 33.0]
Resection,	2 (15.4)	2 (14.3)	1.1 [− 38.4, 41.5]
Metastatectomy	1 (7.7)	1 (7.1)	0.5 [− 36.0, 38.5]
Primary site,	1 (7.7)	1 (7.1)	0.5 [− 36.0, 38.5]
Chemotherapy	11 (84.6)	13 (92.9)	−8.2 [− 46.7, 30.2]
Platinum agent	4 (30.8)	9 (64.3)	−33.5 [− 69.7, 16.3]
ICI	5 (38.5)	10 (71.4)	−33 [− 69.4, 16.6]
Paclitaxel	8 (61.5)	7 (50.0)	11.5 [− 35.6, 53.8]
Radiation	7 (53.8)	8 (57.1)	−3.3 [− 47.7, 42.2]

*CI* confidence interval

^*^Counts not mutually exclusive

**Fig. 3:**
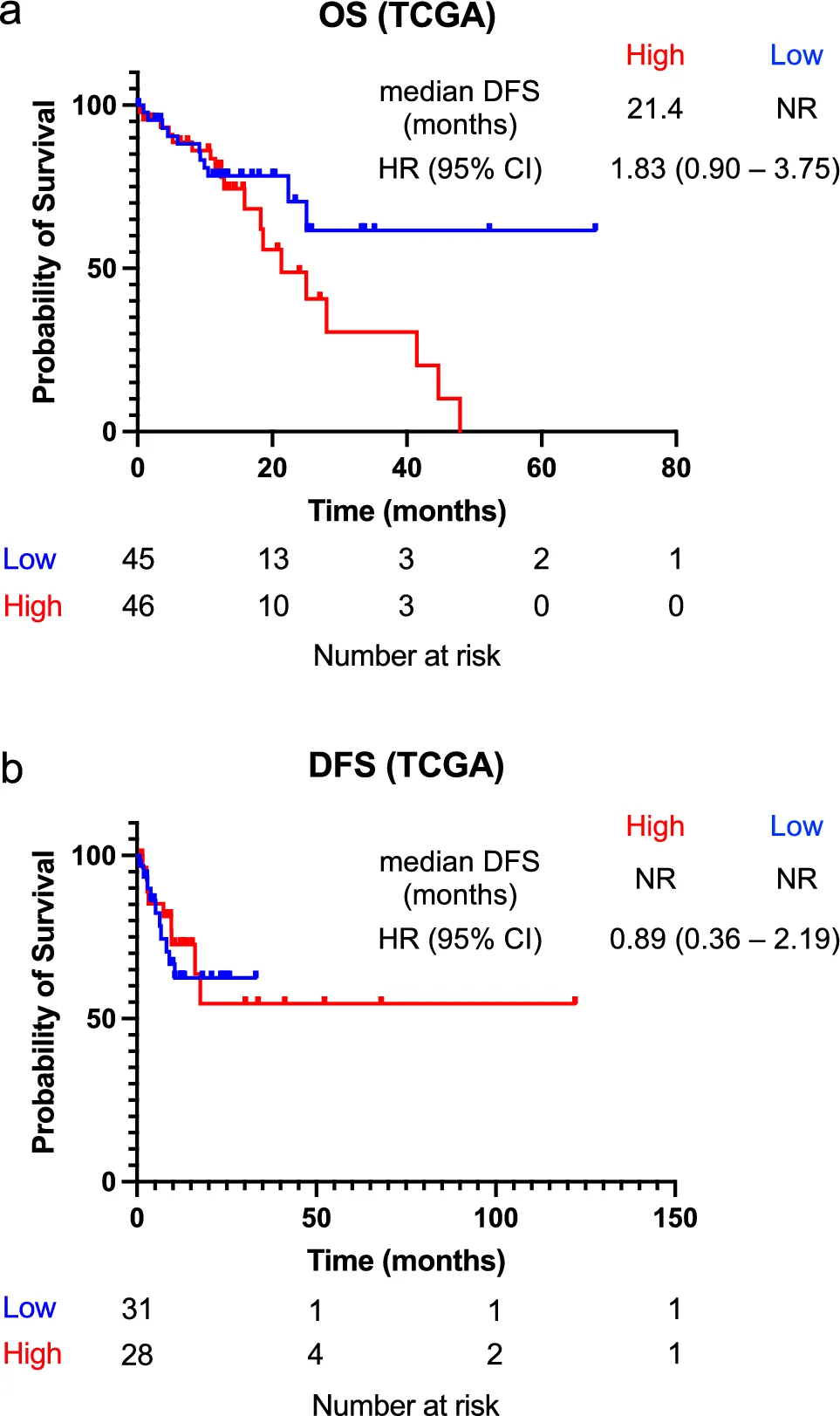
Survival analysis according to *TACSTD2* expression in ESCC from TCGA. **a** Kaplan–Meier curves for OS in patients stratified by *TACSTD2* expression (high vs. low). **b** Kaplan–Meier curves for DFS in patients stratified by *TACSTD2* expression (high vs. low). *DFS* disease-free survival, *ESCC* esophageal squamous cell carcinoma, *NR* not reached, *OS* overall survival, *TACSTD2* tumor-associated calcium signal transducer 2, *TCGA* the cancer genome atlas

In the TCGA cohort, the proportion of patients with a history of smoking (low vs. high: 54.3% vs. 71.7%) and habitual alcohol consumption (low vs. high: 65.2% vs. 80.4%) was higher in the high-expression group than in the low-expression group. In the low-expression group, cT3–4 tumors were more frequently observed (low vs. high: 65.2% vs. 47.8%), whereas lymph node involvement was less common (low vs. high: 67.4% vs. 45.7%). Patients with higher *TACSTD2* expression showed a trend toward poorer OS (HR, 1.83; 95% CI 0.90–3.75; Fig. 3a) than those with lower *TACSTD2* expression. The DFS was comparable between the two groups (HR, 0.89; 95% CI 0.35–2.19; Fig. 3b).

**Fig. 4:**
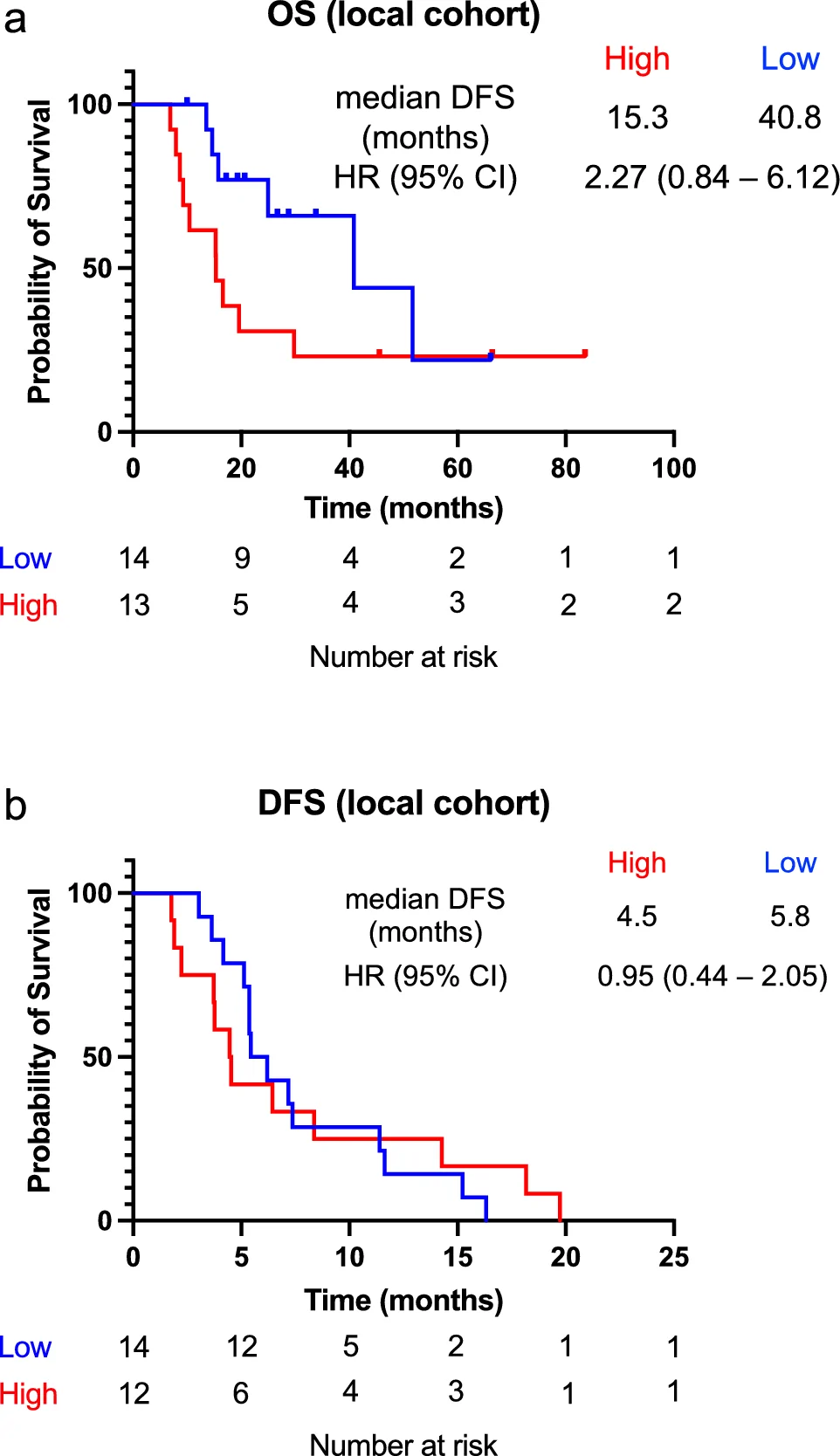
Survival analysis according to *TACSTD2* expression in the institutional cohort. **a** Kaplan–Meier curves for OS in patients stratified by *TACSTD2* expression (high vs. low). **b** Kaplan–Meier curves for DFS in patients stratified by *TACSTD2* expression (high vs. low). *DFS* disease-free survival, *OS* overall survival, *TACSTD2 * tumor-associated calcium signal transducer 2

In the institutional cohort, lymph node involvement was present in all patients in the high-expression group, whereas a subset of patients in the low-expression group was node-negative (low vs. high; 21.4% vs. 0%). Moreover, the high-expression group tended to have a greater number of recurrent sites than the low-expression group (≥ 3 sites: low vs. high, 0% vs. 30.8%). Additionally, platinum-based chemotherapy and immune checkpoint inhibitors (ICIs) were more frequently administered in the low-expression group than in the high-expression group (low vs. high: 64.3% vs. 30.8% and 71.4% vs. 38.5%, respectively). Consistent with findings from the TCGA cohort, the institutional cohort also demonstrated a trend toward worse OS in patients with higher *TACSTD2* expression levels (HR, 2.27; 95% CI 0.84–6.12) but equivalent DFS in the two groups (HR, 0.95; 95% CI 0.44–2.05; Fig. 4a, b).

## Discussion

In this study, we evaluated TROP2/*TACSTD2* expression at both the protein and transcript levels to explore its potential as an ADC target in ESCC. The TCGA cohort provided large-scale copy-number and transcriptome-wide expression analyses in predominantly resectable cases, while the institutional cohort supported protein-level assessment in locally advanced or metastatic disease, including metastatic lesions.

First, our results indicate that TROP2 is strongly and broadly expressed at both primary and metastatic sites in ESCC, supporting its potential as an ADC target. This finding extends previous ESCC reports based predominantly on resected primary tumors to the advanced and metastatic settings most relevant for TROP2-ADC therapy [13, 14]. While staining appeared to be retained after chemotherapy, paired pre-/post-treatment samples were unavailable to confirm within-patient stability. As ADC activity may depend not only on target abundance but also on factors, such as internalization and intracellular trafficking, IHC intensity alone may be insufficient to predict clinical benefit, facilitating the use of more quantitative, compartment-resolved metrics to complement visual IHC assessment. For example, a recent report on NSCLC suggested that a compartment-resolved assessment of membranous and cytoplasmic TROP2 and a normalized membrane ratio (NMR) reflecting membranous versus cytoplasmic TROP2 distribution may better predict clinical outcomes than conventional visual TROP2 IHC scoring [11]. Moreover, given the near-universal, strong TROP2 expression, IHC positivity (positive vs. negative) is unlikely to be useful for patient stratification, underscoring the need for quantitative, compartment-resolved metrics. Future studies on TROP2 expression in ESCC should incorporate such metrics along with clinical outcomes to determine whether they enable patient stratification.

While previous reports characterized TROP2 protein expression in ESCC at the IHC level [13, 14], *TACSTD2* expression and its relationship to gene copy number had not been examined in this disease. To better understand the basis of *TACSTD2* overexpression, we examined CNV and mRNA expression in TCGA. *TACSTD2* amplification was infrequent in ESCC, whereas *TACSTD2* mRNA expression was consistently high across cases and appeared largely independent of copy number status. This observation is consistent with that of a previous study showing that tumor types with high *TACSTD2* mRNA levels generally exhibit high frequencies of strong TROP2 staining by IHC [23, 24]. Experimental studies have suggested that *TACSTD2* expression is primarily regulated at the transcriptional and post-transcriptional levels [25]; however, the exact mechanisms of *TACSTD2* regulation have not been elucidated. Furthermore, we found that *TACSTD2* mRNA in ESCC samples was comparatively higher than that in tumor types, such as NSCLC and BRCA, where TROP2-targeted agents have demonstrated clinical activity. These findings, together with our IHC results, support the idea that ESCC meets the expression-related prerequisites for TROP2-targeted ADCs, warranting further clinical evaluation.

We observed a consistent trend toward poorer OS in patients with higher *TACSTD2* expression in both cohorts, whereas DFS was comparable between the groups. This should be interpreted cautiously given the exploratory, unadjusted nature of our analyses and potential imbalances in baseline characteristics and treatments, although several factors may underlie the discordance between OS and DFS. First, in the institutional cohort, the high-expression group tended to have a greater number of recurrent sites than the low-expression group, suggesting a higher post-recurrence disease burden that can adversely affect OS, despite similar DFS. Second, post-recurrence treatments were not balanced between the groups, with platinum-based chemotherapy and ICIs used more frequently in the low-expression group. The reasons for this imbalance could not be determined. In the TCGA cohort, detailed data on the recurrence site and post-recurrence treatments were unavailable, precluding the assessment of whether differences in post-recurrence disease burden or treatment allocation contributed to the observed OS trend. In addition to these clinical factors, *TACSTD2* may also reflect the tumor biology associated with differential treatment sensitivity. *TACSTD2* has been implicated in the resistance to ICIs in NSCLC [26, 27]. Although this has not been studied in ESCC, such mechanisms can contribute to poorer post-recurrence outcomes and should be examined in larger cohorts. Additionally, nodal involvement was more frequent in the high-expression group in both cohorts, suggesting a potential association between *TACSTD2* expression and lymphatic spread. Similar associations have been reported in gastric and colorectal cancers [28, 29]; however, previous ESCC data have been inconsistent [13, 14]. Therefore, the relationship should be interpreted with caution. Nonetheless, its consistent association with worse OS across the two cohorts raises the possibility that *TACSTD2* expression reflects biologically distinct ESCC subgroups.

This study has some limitations. We assessed membranous and cytoplasmic TROP2 staining using a combined scoring approach, precluding evaluation of the potential biological significance of subcellular localization. Future studies integrating compartment-specific TROP2 staining patterns with *TACSTD2* transcriptional activity, gene copy number status, and clinical outcomes are warranted to further refine the biological and therapeutic relevance of TROP2 in ESCC. Additionally, our institutional cohort was a single-center, retrospective study with a modest sample size. In addition, RNA-seq was conducted only on a subset of the surgical specimens, and the analyses were restricted to tumor tissues because matched normal samples were not available. Analyses were exploratory and unadjusted, and imbalances in treatments and baseline factors may have confounded the survival associations. The two cohorts also differed in stage distribution and clinical annotation, which limits direct quantitative comparison between them. These limitations warrant cautious interpretation and validation in larger adjusted and externally replicated cohorts.

In conclusion, TROP2/*TACSTD2* is robustly and consistently overexpressed in ESCC at both the protein and mRNA levels, despite infrequent *TACSTD2* amplification, supporting its feasibility as a therapeutic target. Across the two cohorts, higher *TACSTD2* expression was suggestive of poorer overall survival, indicating that the high-expression group represents a subgroup with unmet medical need for novel therapeutic strategies. While future studies in larger, externally validated cohorts are needed to establish the prognostic and predictive value of TROP2/*TACSTD2* in ESCC, our findings provide biologic rationale for the ongoing phase II study on sacituzumab govitecan in patients with advanced ESCC has been initiated in Taiwan [30], and for future TROP2-ADC clinical development in this disease.

## Supplementary Information

Below is the link to the electronic supplementary material.

Supplementary file1 (DOCX 31 KB)

Supplementary file2 (DOCX 82 KB)

Supplementary file3 (DOCX 24 KB)

Supplementary file4 (DOCX 258 KB)

## Data Availability

The datasets generated and/or analyzed during the study are not publicly available owing to restrictions of the research ethics protocol but are available from the corresponding author upon reasonable request.
