# Supplementary material for: Tumor-associated calcium signal transducer 2 expression as a promising therapeutic target in esophageal squamous cell carcinoma: a combined the Cancer Genome Atlas and an institutional cohort
Source: Esophagus. 2026 Sep 20;23(4):1003–13. doi: 10.1007/s10388-026-01257-5 (PMC13723502; doi:10.1007/s10388-026-01257-5)
Supplement: Supplementary file 1 [file 10388_2026_1257_MOESM1_ESM.docx]

**Trophoblast cell-surface antigen 2 expression as a promising therapeutic target in esophageal squamous cell carcinoma: a combined The Cancer Genome Atlas and institutional cohort analyses**

***Esophagus***

## Chiaki Inagaki^1,2,^ Hisato Kawakami^1,3*^, Seiichiro Mitani^1^, Hiroshi Iijima^4^, Ryo Matoba^4^, Akihiko Ito^5^, Osamu Shiraisi^6^, Takushi Yasuda^6^, Hidetoshi Hayashi^1^

^1^Department of Medical Oncology, Kindai University Faculty of Medicine, 1-14-1 Miharadai, Minami-ku, Sakai-shi, Osaka, Japan

^2^Genome Medical Center, Kindai University Hospital, 1-14-1 Miharadai, Minami-ku, Sakai-shi, Osaka, Japan

^3^Department of Clinical Oncology, Tohoku University Graduate School of Medicine, Sendai, Japan

^4^DNA Chip Research Inc., 3-1200, Shinmaruko-Higashi, KDX Musashikosugi Blgd. 9F, Nakahara-Ku, Kawasaki-shi, Kanagaaw, 211-0004, Japan

^5^Department of Medicine/Graduate School of Medical Sciences, Kindai University Faculty of Medicine, 1-14-1 Miharadai, Minami-ku, Sakai-shi, Osaka, Japan

^6^Department of Surgery, Kindai University Faculty of Medicine, 1-14-1 Miharadai, Minami-ku, Sakai-shi, Osaka, Japan

Corresponding author’s email address:

kawakami_h@tohoku.ac.jp

Online Resource 1

| **Supplementary Table 1. List of TCGA cancer type abbreviations used in this study.** | |
| --- | --- |
| ACC | Adrenocortical carcinoma |
| BLCA | Bladder urothelial carcinoma |
| BRCA | Breast invasive carcinoma |
| CESC | Cervical squamous cell carcinoma |
| CHOL | Cholangiocarcinoma |
| COAD | Colon adenocarcinoma |
| DLBC | Diffuse large B cell lymphoma |
| ESA | Esophageal adenocarcinoma |
| ESCC | Esophageal squamous cell carcinoma |
| GBM | Glioblastoma multiforme |
| HNSC | Head and neck squamous cell carcinoma |
| KICH | Kidney chromophobe |
| KIRC | Kidney renal clear cell carcinoma |
| KIRP | Kidney renal papillary cell carcinoma |
| LAML | Acute myeloid leukemia |
| LGG | Brain lower grade glioma |
| LIHC | Liver hepatocellular carcinoma |
| LUAD | Lung adenocarcinoma |
| LUSC | Lung squamous cell carcinoma |
| MESO | Mesothelioma |
| OV | Ovarian serous cystadenocarcinoma |
| PAAD | Pancreatic adenocarcinoma |
| PCPG | Pheochromocytoma and paraganglioma |
| PRAD | Prostate adenocarcinoma |
| READ | Rectum adenocarcinoma |
| SARC | Sarcoma |
| SKCM | Skin cutaneous melanoma |
| STAD | Stomach adenocarcinoma |
| TGCT | Testicular germ cell tumors |
| THCA | Thyroid carcinoma |
| THYM | Thymoma |
| UCEC | Uterine corpus endometrial carcinoma |
| UCS | Uterine carcinosarcoma |
| UVM | Uveal melanoma |
