# Supplementary material for: Tumor-associated calcium signal transducer 2 expression as a promising therapeutic target in esophageal squamous cell carcinoma: a combined the Cancer Genome Atlas and an institutional cohort
Source: Esophagus. 2026 Sep 20;23(4):1003–13. doi: 10.1007/s10388-026-01257-5 (PMC13723502; doi:10.1007/s10388-026-01257-5)
Supplement: Supplementary file 2 [file 10388_2026_1257_MOESM2_ESM.docx]

**Trophoblast cell-surface antigen 2 expression as a promising therapeutic target in esophageal squamous cell carcinoma: a combined The Cancer Genome Atlas and institutional cohort analyses**

***Esophagus***

## Chiaki Inagaki^1,2,^ Hisato Kawakami^1,3*^, Seiichiro Mitani^1^, Hiroshi Iijima^4^, Ryo Matoba^4^, Akihiko Ito^5^, Osamu Shiraisi^6^, Takushi Yasuda^6^, Hidetoshi Hayashi^1^

^1^Department of Medical Oncology, Kindai University Faculty of Medicine, 1-14-1 Miharadai, Minami-ku, Sakai-shi, Osaka, Japan

^2^Genome Medical Center, Kindai University Hospital, 1-14-1 Miharadai, Minami-ku, Sakai-shi, Osaka, Japan

^3^Department of Clinical Oncology, Tohoku University Graduate School of Medicine, Sendai, Japan

^4^DNA Chip Research Inc., 3-1200, Shinmaruko-Higashi, KDX Musashikosugi Blgd. 9F, Nakahara-Ku, Kawasaki-shi, Kanagaaw, 211-0004, Japan

^5^Department of Medicine/Graduate School of Medical Sciences, Kindai University Faculty of Medicine, 1-14-1 Miharadai, Minami-ku, Sakai-shi, Osaka, Japan

^6^Department of Surgery, Kindai University Faculty of Medicine, 1-14-1 Miharadai, Minami-ku, Sakai-shi, Osaka, Japan

Corresponding author’s email address:

kawakami_h@tohoku.ac.jp

Online Resource 2

**Consolidated Standards of Reporting Trials diagram of patient selection and sample availability in the local cohort**

ESCC, esophageal squamous cell carcinoma; IHC, immunohistochemistry; TROP2, trophoblast cell-surface antigen 2; WTS, whole transcriptome sequencing
