# Supplementary material for: Tumor-associated calcium signal transducer 2 expression as a promising therapeutic target in esophageal squamous cell carcinoma: a combined the Cancer Genome Atlas and an institutional cohort
Source: Esophagus. 2026 Sep 20;23(4):1003–13. doi: 10.1007/s10388-026-01257-5 (PMC13723502; doi:10.1007/s10388-026-01257-5)
Supplement: Supplementary file 3 [file 10388_2026_1257_MOESM3_ESM.docx]

**Trophoblast cell-surface antigen 2 expression as a promising therapeutic target in esophageal squamous cell carcinoma: a combined The Cancer Genome Atlas and institutional cohort analyses**

***Esophagus***

## Chiaki Inagaki^1,2,^ Hisato Kawakami^1,3*^, Seiichiro Mitani^1^, Hiroshi Iijima^4^, Ryo Matoba^4^, Akihiko Ito^5^, Osamu Shiraisi^6^, Takushi Yasuda^6^, Hidetoshi Hayashi^1^

^1^Department of Medical Oncology, Kindai University Faculty of Medicine, 1-14-1 Miharadai, Minami-ku, Sakai-shi, Osaka, Japan

^2^Genome Medical Center, Kindai University Hospital, 1-14-1 Miharadai, Minami-ku, Sakai-shi, Osaka, Japan

^3^Department of Clinical Oncology, Tohoku University Graduate School of Medicine, Sendai, Japan

^4^DNA Chip Research Inc., 3-1200, Shinmaruko-Higashi, KDX Musashikosugi Blgd. 9F, Nakahara-Ku, Kawasaki-shi, Kanagaaw, 211-0004, Japan

^5^Department of Medicine/Graduate School of Medical Sciences, Kindai University Faculty of Medicine, 1-14-1 Miharadai, Minami-ku, Sakai-shi, Osaka, Japan

^6^Department of Surgery, Kindai University Faculty of Medicine, 1-14-1 Miharadai, Minami-ku, Sakai-shi, Osaka, Japan

Corresponding author’s email address:

kawakami_h@tohoku.ac.jp

Online Resource 3

**Patients’ characteristics of the local cohort**

|  | Surgically resected specimen | Biopsy specimen |
| --- | --- | --- |
| Patients' characteristics | n=40 | n=30 |
| Age, median (range), years | 69(49, 84) | 71(34, 83) |
| Sex,n(%) |  |  |
| Male / Female | 30(75.0) / 10(25) | 20(66.7) / 10(33.3) |
| Smoking history |  |  |
| Yes / No, n(%) | 31(77.5) / 9(22.5) | 22(73.3) / 8(26.7) |
| Havitual drinking |  |  |
| Yes / No, n (%) | 32(80) / 8(20) | 23(76.7) / 7(23.3) |
| Sample site, n (%) |  |  |
| Primary / Metastatic site | 37(92.5) / 3(7.5) | 30(100) / 0 (0) |
| Primary tumor localization, n (%) |  |  |
| Cervical / Thoratic / Abdominal | 6(15.0) / 31(77.5) / 3(7.5) | 1(3.3) / 28(93.3) / 1(3.3) |
| Histology, n (%) |  |  |
| Squamous / Adensquamous | 37(92.5) / 3(7.5) | 30(100) / 0(0) |
| Clinical stage; cStage, n (%) |  |  |
| I - II / III - IV | 5(12.5) / 35(87.5) | 2(6.7) / 28(93.3) |
| cT stage, n (%) |  |  |
| 1-2, 3-4, | 8(20.0) / 32(80.0) | 5(16.7) / 25(83.3) |
| cN stage, n (%) |  |  |
| Negative / Positive | 6(15.0) / 34(85.0) | 1(3.3) / 29(96.7) |
| cM stage |  |  |
| 0, (n, %) | 29(72.5) | 6(20.0) |
| 1; No.104 or 101 alone / other, (n, %) | 11(27.5); 6 (15.0) / 5 (12.5) | 24 (80.0); 4 (13.3) / 20 (66.7) |
| Prior treatment with curative intent*, (n, %) |  |  |
| Yes/No | 37(92.5) / 3(7.5) | 10 (33.3) / 20 (66.7) |
| Chemotherapy | 36 (90.0) | 9 (30.0) |
| Radiation | 4(7.5) | 7(23.3) |
| Resection (including ESD) | 37(92.5) | 3(10.0) |
| Curability, n (%) | n=37 | n=3 |
| R0-1 / R2 | 33 (89.2) / 4(10.8) | 3(100)/ 0(0) |
| Pathological response of neoadjuvant therapy, n (%) | n=36 | n=2 |
| 0 / 1 / 2 / NA | 5 (13.9) / 26 (72.2) / 4 (11.1) / 1 (2.8) | 0 (0) / 0 (0) / 2 (100) / 0 (0) |
| Pathological stage, n (%) | n=36 | n=2 |
| ypStage I- II / III-IV | 10 (27.8) / 26 (72.2) | 1 (50.0) /1 (50.0) |
| Treatment for recurrent/metastatic disease, (n, %) | | |
| Yes/No | 39 (97.5) / 1 (2.5) | 30 (100) / 0 (0) |
| Resection* | 4 (10.0) | 0 (0) |
| Metastatectomy | 3 (7.5) | - |
| Primary site | 2 (5) | - |
| Chemotherapy | 34 (85.0) | 30 (0) |
| Radiation | 21 (52.5) | 12 (0) |
| *Counts not mutually exclusive |  |  |
